# The relationship of compliance with immediate and delayed suggestibility and types of resistant behavioral responses in children aged 10–15 years

**DOI:** 10.3389/fpsyg.2024.1463756

**Published:** 2024-10-04

**Authors:** Gisli Gudjonsson, Valeria Giostra, Tiziana Maiorano, Monia Vagni

**Affiliations:** ^1^Institute of Psychiatry, Psychology and Neuroscience, King’s College London, London, United Kingdom; ^2^Centre for Education and Research in Forensic Psychology, The Department of Humanities, University of Urbino, Urbino, Italy; ^3^Department of Philosophy, Social Sciences, Humanities and Education, University of Perugia, Perugia, Italy

**Keywords:** immediate suggestibility, delayed suggestibility, resistant behavioral responses, ‘direct explanation’ answers, ‘no’ answer, ‘do not know’ answers, source monitoring

## Abstract

This study examined the relationship of compliance with immediate and delayed suggestibility and types of resistant behavioral responses (RBRs) in 454 children (10–15 years) using the Gudjonsson Suggestibility Scale (GSS 2) and a slightly adapted version of the Gudjonsson Compliance Scale (GCS). The GCS was found to have satisfactory internal consistency with this age group. Immediate suggestibility and delayed suggestibility were significantly correlated (small effect size). Compliance was most strongly correlated with Yield 1 (large effect size) and Yield 2 (medium effect size) and only modestly with Shift and delayed suggestibility (both small effect size). Of both theoretical and practical importance was the finding that out of the three resistant behavioral responses (RBRs) where misleading questions were not yielded to, ‘direct explanation’ and ‘no answers’ were the only salient predictors of compliance. ‘Do not know’ answers were found to have the weakest association with compliance. The current findings help better understand the complex relationship of compliance with immediate suggestibility (i.e., Yield and Shift), RBRs, and delayed suggestibility in children. The findings have important implications for future studies as well as interview practice.

## 1 Introduction

Most research with children as witnesses has focused on suggestibility rather than compliance, which is probably mainly due to the absence of a validated compliance scale for children.

Gudjonsson (2003, p. 370) defines ‘compliance’ in an interrogative situation as “the tendency of the individual to go along with propositions, requests or instructions, for some immediate instrumental gain.” The primary drivers behind compliance are avoidance of conflict and confrontation and eagerness to please others, which highlight the psychosocial aspects of compliance.

The GCS was developed primarily for the purpose of detecting vulnerabilities in an interrogative context, particularly in relation to false confessions (Otgaar et al., 2021), but it also measures vulnerability to peer pressure (Sigurdsson and Gudjonsson, 1996) and taking the blame for things they have not done (Gudjonsson et al., 2007). Gudjonsson et al. (2008) found that personal relationship compliance (e.g., requests by family members or friends) and impersonal relationship (e.g., requests by people in authority, such as police, teachers, or salespersons) compliance were different components of the broader psychological construct of compliance as measured by the GCS.

Regarding the term interrogative suggestibility, Gudjonsson and Clark (1986, p. 84) define it as “*the extent to which, within a closed social interaction, people come to accept messages communicated during formal questioning, as the result of which their subsequent behavioral response is affected.*” This definition consists of five components: (i) a social interaction, (ii) a questioning procedure, (iii) a suggestion, (iv) acceptance of the suggestion, and (v) a behavioral response (e.g., answering ‘yes’ to leading questions or shifting answers after interrogative pressure).

These five components were important in the construction of the Gudjonsson Suggestibility Scales (Gudjonsson, 1983, 1984, 1987, 1997), which measure Yield (i.e., giving in to misleading questions before and after interrogative pressure; referred to as Yield 1 and Yield 2, respectively), Shift (i.e., giving in to interrogative pressure), and Total Suggestibility (i.e., Yield 1 and Shift added together).

In the original validation study of 119 adults, compliance correlated significantly with Yield 1 (*r* = 0.40), Shift (*r* = 0.53), and Total Suggestibility (*r* = 0.55). In a subsequent study involving court referrals, Gudjonsson (1990) found that intellectual skills, acquiescence, suggestibility, and compliance loaded on three separate factors with compliance (−0.81) and total suggestibility (−0.67) loading heavily on the third factor relatively independent of the IQ subtests and acquiescence. Based on the findings from the two studies, Gudjonsson (2003) concluded that suggestibility and compliance represent overlapping constructs, each with their individual characteristics. The main difference was that suggestibility relied on the acceptance of a suggestion, whereas no private acceptance was required for compliance. The main driver for interrogative suggestibility, in contrast to compliance, was seen as a failure in discrepancy detection (i.e., a source monitoring problem).

However, suggestibility, as measured by the Gudjonsson Suggestibility Scales, is multifaceted and Polczyk et al. (2024) point out that some Yield responses may be driven by compliance, rather than source monitoring problems. This fits in with the Gudjonsson and Clark (1986) broad definition of interrogative suggestibility that acceptance of a suggestion (i.e., immediate suggestibility) does not necessarily mean that the person subsequently incorporates it into their memory recollection (i.e., delayed suggestibility). The latter involves a different process where source monitoring difficulties are at the forefront.

Since the publication of the GSS Manual (Gudjonsson, 1997), two separate measures have been added to the GSS. First, ‘delayed suggestibility’ (i.e., the number of erroneous items from the misleading questions on the GSS that are incorporated into memory recall at 1-week follow-up) (Vagni et al., 2015; Gudjonsson et al., 2020), and second, the type of ‘resistance behavioral responses’ (RBRs; i.e., ‘do not know’, ‘no’, and ‘direct explanation’ answers) that makeup Yield 1 and Yield 2 (Gudjonsson and Young, 2021; Gudjonsson et al., 2021, 2022; Polczyk et al., 2024; Wachi et al., 2019).

Previous research has shown that immediate suggestibility and delayed suggestibility are only modestly correlated (Gudjonsson et al., 2022; Wachi et al., 2019; Polczyk et al., 2024). We expect this also to be the case in the current study. The main theoretical reasoning for investigating delayed suggestibility and RBRs in relation to compliance is that delayed suggestibility and DK answers have a weak relationship with compliance due to their firm link with source monitoring problems (Gudjonsson, 2003). In contrast, direct explanation answers for rejecting the misleading suggestion are therefore a clear indication of effective source monitoring and the ability to verbally articulate and reject a suggestion with an explanation.

In this study, we explore the suitability and internal reliability of a slightly adapted version of the adult standardized Gudjonsson Compliance Scale (GCS; Gudjonsson, 1989, 1997) with two age bands (10–12 and 13–15 years). We then correlate the compliance scores in each age band with all the administered measures on the Gudjonsson Suggestibility Scale (GSS), which is comprised of verbal memory, confabulation, yielding to misleading questions, changes in answers following negative feedback, resistant behavioral responses (do not know’, ‘no’, and ‘direct explanation’ answers), and delayed suggestibility. To explore the most powerful suggestibility predictors of compliance after controlling for age, sex, memory, and confabulation, linear regression models were used. This will hopefully provide a more holistic picture of the subtle relationship between compliance and suggestibility (Polczyk et al., 2024).

In a recent Chinese study (Hang et al., 2024), a validation of an adapted version of the GCS was used for participants down to age 14 years. The current study goes further than previous studies and provides an important and much-needed addition to the literature.

Despite the absence of studies into compliance, as measured by the GCS, among children, there are reasonable grounds for the following hypotheses:

Hypothesis 1: With minor adaptions, the GCS can be used reliably with children down to the age of 10 years.

Hypothesis 2: All the suggestibility scores (i.e., Yield 1, Yield 2, Shift, and Total Suggestibility) will correlate significantly with compliance in the two age bands (10–12 and 13–15 years).

Hypothesis 3: Immediate and delayed suggestibility will be significantly correlated, but with small effect size.

Hypothesis 4: Of the three resistance behavioral responses (RBRs), ‘direct explanation’ and ‘no’ answers are better predictors of compliance than ‘do not know’ answers.

## 2 Materials and methods

### 2.1 Participants

The sample included 454 participants (258 males, 56.8%; 196 females, 43.2%) aged between 10 and 15 years (*M* = 12.14 and *SD* = 1.75) and mean IQ = 98.8 (min–max = 80–118; SD = 9.04). The sample was selected randomly from several Italian schools after collecting informed consent signed by parents.

The exclusion criteria followed were as follows: (a) Children with visual and hearing impairments; (b) foreign children with poor understanding of the Italian language (indication provided by the teachers or detectable during the administration of the tests); (c) Children with an IQ score below 80; and (d) protocols with missing responses. The cut-off IQ at 80 (low average range) was used as the GCS requires a reasonable IQ for understanding the 20 items of the questionnaire, unlike the GSS (Gudjonsson, 1997).

The participants were met after their parents/guardians submitted signed consent forms.

The participants were categorized into two bands, each: 10–12 years (*N* = 271), and 13–15 years (*N* = 183) to explore differences between the two age bands. This also provides separate norms for the two age bands for forensic evaluations.

### 2.2 Instruments

#### 2.2.1 Gudjonsson suggestibility scales

The Gudjonsson Suggestibility Scale (GSS 1 and GSS 2; Gudjonsson, 1997) is a validated instrument for measuring immediate and delayed suggestibility in children aged 7 to 16 years (Gudjonsson et al., 2016; Vagni et al., 2023). It is comprised of a short story that is read out to the participant, he/she provides immediate recall, there is then a delay of approximately 50 min, after which delayed recall is typically obtained, followed by 20 questions, 15 of which are misleading. These measure susceptibility to misleading questions. This provides Yield 1, which is followed by negative feedback, after which the 20 questions are repeated, giving Yield 2 and Shift.

In contrast to the standard administration procedure (Gudjonsson, 1997), following the method standardized by Gudjonsson et al. (2016), the delayed recall was not measured before the suggestive interview, but after a week, providing delayed recall at 1-week follow-up (Singh and Gudjonsson, 1984) and the additional measurement of delayed suggestibility (Gudjonsson et al., 2016). Resistant behavioral responses to misleading questions (Gudjonsson et al., 2021, 2022) were also measured (see Table 1 for a summary of all the ‘standard’ and ‘additional’ measures).

**Table 1:** The GSS Manual (Gudjonsson, 1997) the ‘standard’ and ‘additional’ measures.

Standard GSS measures for immediate suggestibility*	Description
Immediate recall [IR]	Number of items correctly recalled immediately after the story has been read out to the participant. [Max. 40].
Delayed recall [DR] as per standard procedure	Number of items correctly recalled after 50 min [Max. 40].
Confabulation on [IR]	Number of distortions and fabrications in immediate recall.
Confabulation on [DR]	Number of distortions and fabrications in delayed recall.
Yield 1 suggestibility	The number of misleading questions yielded to *prior to* negative feedback. [Max. 15].
Yield 2 suggestibility	The number of misleading questions yielded to *after* negative feedback. [Max 15].
Shift	The number of answers tangibly changed after negative feedback. [Max. 20].
Total suggestibility	Yield 1 and Shift added together to give an overall level of suggestibility. [Max. 35].

Additional measures**	Description
Delayed Recall [DR] at follow-up	Number of items correctly recalled at 1-week follow-up [Max. 40] (Singh and Gudjonsson, 1984).
Confabulation at follow-up	Number of distortions and fabrications in delayed recall at 1-week follow-up (Smith and Gudjonsson, 1995).
Delayed suggestibility	Number of erroneous items from the misleading questions incorporated into memory recall at 1-week follow-up (Gudjonsson et al., 2016).
Resistant behavioural responses (RBRs)	When asked misleading unanswerable questions interviewees can resist yielding to the suggestion in three main ways: Giving a ‘no’ reply answer to the question without a direct explanation as to why they cannot answer it (‘NO’ answers). [Max. 15]. Giving ‘do not know’ or ‘not sure’ (‘DK’) answers, which is the weakest RBR, because it could indicate a problem with ‘source monitoring’ (i.e., failure to identify the discrepancy between what they observed and that subsequently suggested to them by the interviewer or others) (Gudjonsson, 2018). Stating that what was suggested is factually wrong (e.g., ‘that was not mentioned’ or ‘that did not happen’), referred to as ‘direct explanation’ (‘DE’) answers. [Max 15].

*Gudjonsson (1997). **Vagni et al. (2015), Vagni et al. (2023), Gudjonsson et al. (2022), and Gudjonsson and Young (2021).

The current sample had acceptable internal consistency for the GSS 2 scores (Cronbach’s alpha coefficient: Yield 1, *α* = 0.82; Yield 2, *α* = 0.82; Shift, *α* = 0.70, and Total Suggestibility, *α* = 0.78). The internal reliability coefficients in the current study are like those of a previous and larger Italian sample (Gudjonsson et al., 2016).

The Italian GSS 2 translation has already been used in several studies involving children of different ages and with intellectual disabilities (Vagni et al., 2015, 2018, 2021, 2022, 2023, 2024a; Gudjonsson et al., 2020, 2021, 2022).

#### 2.2.2 Gudjonsson compliance scale (GCS)

The GCS consists of 20 statements to which the participant must answer true or false (Gudjonsson, 1989, 1997). It was developed to complement the measurement of interrogative suggestibility within a questioning context. Suggestibility and compliance were seen as overlapping constructs but driven by different mechanisms. Polczyk et al. (2024) argue that compliance involves a social mechanism, not a cognitive one. This is the position we take in the current study.

Suggestibility was seen to be primarily influenced by belief systems and failure in source monitoring, whereas compliance was seen as involving a conscious effort to “go along with propositions, requests or instructions, for some instrumental gain” (e.g., eagerness to please and avoidance of conflict and confrontation) (Gudjonsson, 2003, p. 370). In the original study, the alpha coefficient for the 20 items tested on a sample of adults was 0.71 and the test–retest correlation, l-3 months apart, was 0.88 (*p* < 0.001).

To make the original adult version of the GCS more suitable for children, in the current study, the following items were adapted with some minor changes: 1, 3, 4, 5, 6, 8, and 12. The sentences present simpler language without having changed their meaning. For example, in Item 4 some examples were added to explain the expression “people in authority.” The order of succession of the items remained unchanged from the standardized version for adults. The modified items are provided in the Appendix Table A1.

#### 2.2.3 Raven progressive matrices

This is a non-verbal measure of intellectual abilities. Raw scores are converted to percentiles with the corresponding IQ values by age range. Participants under the age of 12 completed the Colored Progressive Matrices which consists of 36 items (CPM; Raven, 1984; Belacchi et al., 2008). Participants of 12 years and above completed the Standard Progressive Matrices, consisting of 60 items (Raven, 1954).

### 2.3 Procedure

The GSS 2 was administered following the same procedure in the two age band groups. This followed the standard procedure (Gudjonsson, 1997), except for delayed recall. The GSS 2 questions were administered after the Raven’s Matrices which took between 40 and 50 min without the standard delayed recall being obtained. Delayed recall and delayed suggestibility were obtained after 1 week, along with the GCS.

All the GSS scores used were tape-recorded and then transcribed for accuracy purposes.

The ethics committee of the University of Urbino approved the study with specific recruitment of children (Minute no. 28 of 18 March 2020). The ethical guidelines of the Declaration of Helsinki were followed and respected in the study.

### 2.4 Analytical strategy

Cronbach’s alpha coefficient was used to measure the internal consistency of 20 GCS items. This helps to determine whether the 20 items consistently measure the same characteristic. Cronbach’s alpha quantifies the level of agreement on a standardized 0 to 1 scale (Taber, 2018).

Means with their standard deviations were provided for continuous variables. Pearson correlations were performed to investigate the association between variables. Age-band *t*-test comparisons were conducted on all variables of interest. We used Cohen’s *d* (Cohen, 1992) to measure effect sizes regarding the differences between the two age bands (*t*-tests): 0.20, 0.50, and 0.80 were used to detect small, medium, and large effect sizes, respectively. The corresponding Cohen’s *d* effect sizes regarding the correlation coefficients were 0.10, 0.30, and 0.50.

To investigate the relative contribution of behavioral resistant responses (RBRs: NO, DK, and DE answers), Shift and delayed suggestibility on GCS compliance, two hierarchical regression models were performed with compliance as the dependent variable. In Model 1 age, sex, immediate recall, and confabulation on immediate recall were entered in Step 1, with NO 1, DK 1, and DE 1. Shift suggestibility and delayed suggestibility were added in Step 2 to measure their unique contribution to the variance in compliance. In Model 2 age, sex, delayed recall, and confabulation on delayed recall were entered in Step 1. Shift, delayed suggestibility, NO 2, DE 2, and DK 2 were added in Step 2. This allowed a comparison between the effects of different behavioral responses for Yield 1 and Yield 2, respectively.

## 3 Results

### 3.1 Internal consistency of the 20 GCS items

**Figure 1:**
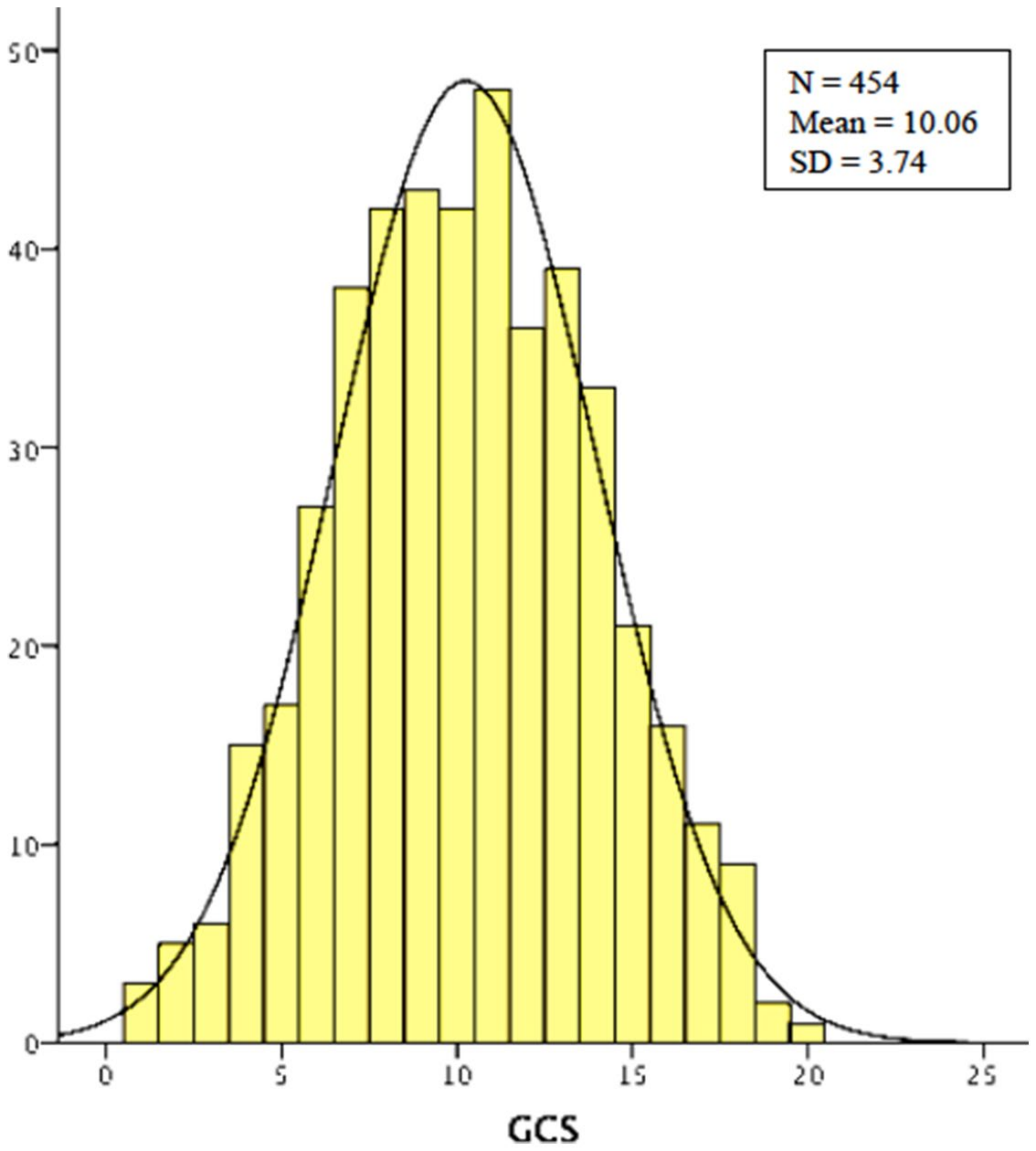
Sample distribution for GCS scores.

The Cronbach’s alpha coefficient was used to measure the internal consistency (reliability) of the 20 GCS items for the individual age bands and then for the total sample. This helped determine whether the GCS items consistently measure the same characteristic. The level of agreement on a standardized scale of 0 to 1 is shown in Table 2. Figure 1 shows the sample distribution for GCS scores, which represents a reasonably normal curve.

**Table 2:** Cronbach’s alpha for the two age bands individually and total sample.

Age band	*N*	Cronbach’s alpha
10–12 years	271	0.69
13–15 years	183	0.73
Total sample	454	0.73

The Cronbach alpha for the total sample and two age bands, individually, is satisfactory with a slight dip in the 10–12-year group (Taber, 2018).

### 3.2 Age-band differences in GCS and GSS 2 scores

Table 3 shows the differences in the GCS and GSS 2 mean scores between the two age bands. Only differences of *p* < 0.01 and *p* < 0.001 were highlighted in the Table. In terms of effect sizes, the strongest age effects were for compliance, DK 1 and DK 2 replies, and Yield 1. The lower age band had a significantly higher IQ than the older age band, but the effect size is small. This small difference is unlikely to be important to the findings of the main study, but it represents a limitation. It is unclear why this two-point IQ score difference exists in the current study.

**Table 3:** Descriptive statistics for all sample and age groups, *t*-tests, and effect sizes (N = 454).

Age band	All sample *N* = 454	10–12 years *N* = 271	13–15 years *N* = 183	*t*	*d*
	Mean (SD)	Mean (SD)	Mean (SD)		
GCS	10.06 (3.74)	11.04 (3.59)	9.07 (3.88)	5.71**	0.54
IQ	98.77 (9.04)	100.90 (8.86)	98.63 (9.21)	2.64*	0.25
Immediate recall (IR)	13.78 (5.46)	14.39 (5.35)	13.17 (5.56)	2.35	0.22
Delayed recall (DR)	10.61 (9.49)	11.22 (10.41)	9.61 (7.67)	1.68	0.18
Confabulation on IR	1.03 (1.18)	1.01 (1.20)	1.05 (1.16)	−0.39	0.03
Confabulation on DR	1.07 (1.18)	1.17 (1.25)	0.90 (1.05)	2.20	0.23
Yield 1	7.54 (3.52)	8.14 (3.43)	6.93 (3.60)	3.59**	0.34
Yield 2	8.49 (5.51)	8.99 (7.24)	7.99 (3.77)	1.70	0.17
Shift	5.52 (3.23)	5.40 (3.26)	5.63 (3.20)	−0.76	0.07
Total suggestibility	13.06 (5.40)	13.54 (5.48)	12.58 (5.32)	1.83	0.18
Delayed suggestibility	0.71 (0.96)	0.77 (1.03)	0.62 (0.83)	1.56	0.16
RBRs					
NO 1	4.68 (2.38)	4.85 (2.37)	4.51 (2.38)	1.50	0.14
NO 2	4.11 (2.26)	4.30 (2.20)	3.92 (2.31)	1.75	0.17
DE 1	1.86 (2.96)	1.52 (2.74)	2.20 (3.18)	−2.41	0.23
DE 2	1.82 (3.18)	1.72 (3.13)	1.91 (3.22)	−0.63	0.10
DK 1	0.93 (1.76)	0.54 (1.45)	1.32 (2.07)	−4.77**	0.44
DK 2	0.80 (1.85)	0.45 (1.44)	1.15 (2.26)	−4.00**	0.37

*p < 0.01, **p < 0.001. GCS, Gudjonsson Compliance Scale (*p* < 0.05 was not reported as the focus was on the most salient age group differences); NO 1 and 2 = simple no answers; DE 1 and 2 = direct explanation answers; DK 1 and 2 = do not know answers.

### 3.3 Correlations of GCS with IQ and GSS 2 scores

Table 4 shows the correlations of GCS with IQ and the GSS 2 scores, for each age band separately, and the total sample. Overall, there was a reasonable consistency in the correlations across the two age bands, which is reassuring.

**Table 4:** Pearson Correlations of the GCS score with IQ and all the GSS 2 scores for the total sample and the two age groups individually.

Variables	Total sample *N* = 454	10–12 years *N* = 271	13–15 years *N* = 183
IQ	−0.032	−0.081	−0.047
Immediate recall	−0.137**	−0.125*	−0.236**
Delayed recall	−0.073	−0.120*	−0.059
Confabulation 1	0.014	0.016	0.024
Confabulation 2	0.102*	0.144*	−0.068
Yield 1	0.501***	0.460***	0.508***
Yield 2	0.198***	0.134*	0.352***
Shift	0.169***	0.218***	0.137*
Total suggestibility	0.428***	0.418***	0.430***
Delayed suggestibility	0.122*	0.086	0.145*
RBRs			
NO 1	−0.221***	−0.281***	−0.202**
NO 2	−0.110*	−0.168**	0.094
DE 1	−0.329***	−0.306***	−0.320***
DE 2	−0.282***	−0.296***	−0.269***
DK 1	−0.150**	−0.064	−0.135*
DK 2	−0.114*	0.005	−0.138*

*p < 0.05; **p < 0.01; ***p < 0.001. NO 1 and NO 2 = no answers; DE 1 and 2 = direct explanation answers; DK 1 and 2 = do not know answers.

The GCS score correlated significantly with all the immediate suggestibility scores, ranging from small to large effect sizes. The highest correlation was between compliance and Yield 1 for both age bands, followed by total suggestibility. Regarding the three different RBRs, compliance was most highly (negatively) correlated with DE 1 (large effect size) and DE 2 (medium effect size).

Compliance was not significantly correlated with IQ and had a low negative correlation with immediate recall (small effect size).

Compliance only correlated significantly with delayed suggestibility in the older age group and the correlation was small (*r* = 0.145, *p* < 0.001).

Delayed suggestibility was significantly correlated with Confabulation 1 (*r* = 0.158, *p* < 0.001) and Confabulation 2 (*r* = 0.181, *p* < 0.001) (see Appendix Table A2).

Delayed suggestibility was significantly correlated with Yield 1 (*r* = 0.224, *p* < 0.001), Yield 2 (*r* = 0.122, *p* < 0.05), and Total Suggestibility (*r* = 0.177, *p* < 0.001), but not with Shift (*r* = 0.049, ns).

Regarding the relationship between delayed suggestibility and type of resistant behavioral responses (RBRs), delayed suggestibility correlated negatively with DE 1 (*r* = −0.123, *p* < 0.05) and DE 2 (*r* = −0.152, *p* < 0.01), but not with DK answers. Delayed suggestibility correlated negatively with NO 1 answers (*r* = −0.108, *p* < 0.05) but not with NO 2 answers (*r* = −0.061, ns).

### 3.4 Multiple regressions

The results of the two hierarchical regression models are shown in Table 5. VIF values were calculated: in the first model all predictors had values between 0.802 and 1.247 and for the second model between 0.797 and 1.254. This shows little multicollinearity in the data.

**Table 5:** Hierarchical linear regression models of the age, sex, immediate and delayed recall, confabulation 1 and 2 and RBRs on GCS (*n* = 454).

	Model 1					Model 2				
	Predictor	*B*	*β*	CI lower	CI upper	Predictor	*B*	*β*	CI lower	CI upper
Step 1	Intercept	19.38		16.27	22.48	Intercept	17.34		14.31	20.37
	Age	−0.64	−0.28***	−0.85	−0.43	Age	−0.60	−0.27***	−0.81	−0.39
	Sex	0.39	0.05	−0.34	1.16	Sex	0.26	0.03	−0.48	0.99
	IR	−0.14	−0.19***	−0.20	−0.07	DR	−0.04	−0.10*	−0.80	−0.01
	Confabulation on IR	0.02	0.01	−0.33	0.28	Confabulation on DR	0.24	0.07	−0.07	0.55
	*R^2^ = 0.108*		*F* = 12.441***			*R^2^ = 0.088*		*F* = 9.912***		
Step 2	Intercept	19.99		16.85	23.13	Intercept	18.26		15.01	21.51
	Age	−0.52	−0.23***	−0.72	−0.32	Age	−0.57	−0.25***		
	Sex	0.02	0.01	−0.66	0.70	Sex	0.09	0.01	−0.62	0.79
	IR	−0.02	−0.03	−0.09	0.05	DR	−0.01	−0.03	−0.05	0.03
	Confabulation on IR	−0.07	−0.02	−0.35	0.22	Confabulation on DR	0.28	0.08	−0.03	0.58
	Shift	0.04	0.03	−0.08	0.15	Shift	0.05	0.04	−0.07	0.17
	Delayed suggestibility	0.10	0.02	−0.26	0.45	Delayed Suggestibility	0.13	0.03	−0.25	0.50
	NO 1	−0.50	−0.31***	−0.66	−0.36	NO 2	−0.28	−0.16**	−0.44	−0.11
	DE 1	−0.44	−0.33***	−0.57	−0.32	DE 2	−0.33	−0.26***	−0.46	−0.21
	DK 1	−0.22	−0.09*	−0.41	−0.02	DK 2	−0.16	−0.05	−0.31	0.08
	*R*^2^ = 0.260	ΔR^2^ = 0.151***	*F* = 15.777***			*R*^2^ = 0.180	ΔR^2^ = 0.092***	*F* = 9.880***		

*p < 0.05; **p < 0.01, ***p < 0.001; Sex: 1 = male; 2 = female; IR = immediate recall; DE1 and 2 = direct explanation answers; DK 1 and 2 = do not know answers.

Table 5 shows that the variables in Step 1 accounted for 11.1 and 8.8% of the variance in compliance in the two models, respectively, which largely comprised of younger age and poorer immediate and delayed recall. The variables inserted in Step 2 added 15.1 and 9.2% to the variance in compliance, respectively. The variance explained in step 2 was given by the Resistant Behavioral Responses, and in particular by the “direct explanations” (DE1 and DE2) and by the “no” responses (NO1 and NO2). As the three RBRs made up all of Yield 1 and Yield 2, they were left out of the regression due to the high multicollinearity in the combined Yield and RBRs data.

## 4 Discussion

The current findings support Hypothesis 1 that the GCS, with slightly adapted wording to make it more applicable to children, can be reliably used with children down to the age of 10 years. The Cronbach’s alpha obtained for the two age bands in the current study is consistent with those obtained in adult groups during the initial validation (Gudjonsson, 1989). Of course, Cronbach’s alpha has its limitations in determining homogeneity (Taber, 2018), but it serves the purpose of the current study. Future studies with children could use a more detailed analysis of the psychometric properties of the GCS (Hang et al., 2024).

Compliance was significantly correlated with all the suggestibility scores, supporting Hypothesis 2. However, unlike the findings with adults (Gudjonsson, 1989), Yield 1 was more strongly correlated with compliance than Shift. In the current study, compliance and Yield 1 shared 25% of the variance with compliance. In contrast, the shared variance of compliance with Shift and Yield 2 were 4 and 3%, respectively (small effect sizes).

This may suggest a more complicated relationship between compliance and Shift in children than adults. According to the Gudjonsson and Clark (1986) model of interrogative suggestibility, Yield and Shift are thought to be primarily driven by the coping strategies that are generated and implemented during questioning. Whereas Yield is heavily influenced by the extent to which interviewees can utilize resistant behavioral responses (RBRs) when dealing with uncertainty, expectation and trust, Shift is more influenced by the ability to cope with interpersonal pressure, linking it theoretically more to compliance (Gudjonsson, 2003).

The relationship between GCS compliance and coping strategies seems more straightforward than with Shift. This is due to Shift representing both an increase in yielding to misleading questions (Yield 2) following the negative feedback, which is the typical response, whilst it sometimes leads to increased strategic coping (i.e., deliberate, active, and goal-directed) and reduced Yield 2 (Gudjonsson, 1995).

Gudjonsson and Sigurdsson (2003) found that the two best predictors of compliance were low self-esteem (i.e., how we perceive and value ourselves) and denial coping (i.e., avoiding facing uncomfortable thoughts, situations, or emotions). Both added similarly to the variance in compliance and when combined accounted for 25% of the total variance (i.e., large effect size).

Morgan et al. (2020) assessed GSS suggestibility and GCS compliance both before and after highly stressful mock interrogations in a military setting. The main findings were that the stress-induced interrogation paradigm increased Shift, but not Yield 1, and compliance. Compliance increased more after the mock interrogation when dissociation levels (i.e., frequency and intensity of dissociation symptoms) were high at baseline testing, suggesting a moderating effect of pre-interrogation dissociation on compliance. The finding in the Morgan III et al. study suggests that dissociation is a moderating variable between situational distress during interrogation and compliance. This is a novel finding that merits further research. It shows the complicated relationship between Shift and compliance in a stressful interrogation setting, highlighting the importance of different mediating factors.

In our study, younger children showed a higher score on compliance, and this seems to satisfy the theoretical construct of the GCS according to which people are more compliant to avoid conflicts and not to oppose authoritative figures (see Table 3). For younger children, adults can be perceived as more powerful authority figures, and this may lead them to be more likely to comply with their requests and demands. According to previous studies, younger children showed higher immediate suggestibility and less ability to give more cognitively complex resistant responses, such as direct explanation and do not know answers (Gudjonsson et al., 2016, 2021, 2022; Waterman and Blades, 2011; Vagni et al., 2024b). With age, children increase their cognitive skills of source monitoring and cope more appropriately and confidently with suggestive interviews (Vagni et al., 2023).

Hypothesis 3 was mainly supported. Immediate suggestibility(i.e., Yield 1, Yield 2, and Total Suggestibility, but not Shift) and delayed suggestibility were significantly correlated, but with small effect size. The study broadly supports the findings of Gudjonsson et al. (2022), Wachi et al. (2019), and Polczyk et al. (2024). The absence of a significant relationship between delayed suggestibility and Shift in the current study is not surprising due to Shift being primarily associated with interrogative pressure, hence psychosocial factors, rather than the source monitoring factors more typically associated with Yield (Gudjonsson, 2018).

In the current study, delayed suggestibility was more strongly associated with confabulation than immediate suggestibility, which suggests a stronger relationship between delayed than immediate suggestibility with a source monitoring mechanism. However, as Polczyk et al. (2024) point out, some Yield responses may be driven by compliance, rather than the source monitoring problems, which suggests that both cognitive and psychosocial factors are relevant to fully understanding interrogative suggestibility. The implication is that compliance, as measured by the GCS, and suggestibility, as measured by the GSS (Forms 1 and 2) measure overlapping constructs both of which are relevant to suggestive police interviews.

Whereas avoidance/denial coping appears to be a likely mechanism for both immediate suggestibility (Gudjonsson, 1988) and compliance (Gudjonsson and Sigurdsson, 2003), it does not appear to be related to delayed suggestibility (Maiorano and Vagni, 2020). One likely factor is that psychosocial factors exacerbate immediate suggestibility and compliance but not delayed suggestibility (Ridley and Gudjonsson, 2013).

In real-life criminal cases of internalized false confessions, delayed suggestibility (i.e., incorporating suggested information into one’s belief system and memory), typically requires time delay and reduction in stress and interpersonal (e.g., good cop and bad cop routine) and environmental (e.g., being isolated in a police cell) factors supporting the false memory. The case of Stephen Miller, one of the ‘Cardiff Three’, is a good example of the source monitoring process involved (Gudjonsson, 2018, pp. 39–45).

After seven police interviews consisting of relentless pressure, Interview 8 shows how Miller begins to doubt his own memory stating, “I could have been there” (i.e., when his ex-girlfriend was murdered), followed by him crying and expressing doubts about his memory, seeking information from the officers about what he supposedly did, and then in a later interview falsely admitting to having participated in stabbing the victim. This breakdown in ‘reality [source] monitoring’ was evident from listening to the police interview tapes, and confirmed by a subsequent clinical interview (Gudjonsson, 2018, 2022).

Supporting Hypothesis 4, the linear multiple regression models in Table 5 showed that out of the three RBRs for both the first and second suggestive interviews (Yield 1 and Yield 2), after adjusting for age, sex, recall, and confabulation, ‘direct explanation’ (*β* = −0.33; *β* = −0.26) and ‘no’ (*β* = −0.31; *β* = −0.16) answers were better predictors of compliance than ‘do not know’ (*β* = −0.09; *β* = −0.05) answers. The implication is that the relationship between Yield and compliance is influenced by the type of RBR with DK answers having the weakest link with compliance, leading to susceptibility to delayed suggestibility (Gudjonsson, 2018). It is relevant here that the first-ever study into the GSS (Gudjonsson, 1983) found that rated lack of confidence in memory was significantly associated with Yield type suggestibility but not with Shift.

The current findings provide an example of the subtle relationship between compliance and suggestibility (Polczyk et al., 2024). Direct explanation answers are clearly most robustly associated with compliance and do not know answers the least. The former provides a clear indication of effective source monitoring and discrepancy detection as well as the ability to articulate a reasoned response. This requires both cognitive processing and a challenging behavioral response to the interviewer (i.e., a psychosocial response).

Regarding the relationship between delayed suggestibility and the type of resistant behavioral responses (RBRs), delayed suggestibility correlated negatively with DE 1 and DE 2. DE answers, followed by No answers, are less likely to be associated with source monitoring problems than DK answers. This suggests that to fully understand vulnerabilities during questioning, police interviewing experts need to have a more sophisticated understanding of what RBRs mainly contribute to the Yield 1 and Yield 2 scores.

The current findings raise important issues about the relationship of source monitoring in relation to Yield, different RBRs, Shift and compliance. Polczyk et al. (2024) suggest that future studies could combine the RBR paradigm with a source monitoring questionnaire. We agree. In addition to using source monitoring measures, following the findings of Morgan et al. (2020), pre-interrogation measures of dissociation might also prove helpful in refining the differential mechanisms for immediate and delayed suggestibility and compliance and the different processes involved.

Vagni et al. (2023) tested the suggestibility of 128 children, aged between 10 and 15 years, twice, 6-months apart using the GSS 2 and GSS 1, respectively. There was a significant reduction in Yield 1 and Yield 2 suggestibility (small effect size) and an increase in confabulation (large effect size; Cohen’s *d* = 0.85) on the second testing whilst no significant change was noted on Shift. The implication is that with repeated testing older children learn to cope better with leading questions, but not with interrogative pressure.

This may partly help explain the relatively low correlation between compliance and Shift in the current study. It corroborates Gudjonsson’s (2003) review of the available evidence that children remain particularly vulnerable to psychosocial and interrogative pressure into adulthood, whereas they learn in early adolescence how to cope with leading questions with DE replies increasing with age in adolescence. In future research, it is also important to investigate the relationship between RBRs and autobiographical skills in children (Vagni et al., 2024a).

The main limitation of the current study is that it only focuses on two age bands of children. We recommend that the current study be replicated in older children (16–17), younger adults (18–25), and adults over 25 years. This might clarify the possible age effect on the relationship of compliance with immediate and delayed suggestibility and RBRs.

## Data Availability

The raw data supporting the conclusions of this article will be made available by the authors, without undue reservation.

## Appendix

**Table A1:** – Original and modified items of the GCS for Children.

Original items	Modified items 1, 3, 4, 5, 6, 8, 12
1. As a child I always did as my parents told me	1. I always do as my parents tell me
3. I am not too concerned what people think of me	3. I am not very worried about what others think of me
4. I tend to become easily alarmed and frightened when I am in the company with people in authority	4. I tend to get alarmed and easily worried when I am with people in authority (school principal, teacher, policeman, ‘director’, etc.)
5. When I was a child, I sometimes took blame for things I had not done	5. I have sometimes taken blame for things I had not done
6. When I am uncertain about things, I tend to accept what people tell me	6. When I am not sure, I tend to accept what others tell me
8. I would describe myself as a very obedient person	8. I am very obedient
12. I try very hard not to offend people in authority	12. I try not to offend people in authority (school principal, teacher, policeman, director, etc.)

**Table A2:** Pearson correlations between GSS2 scores, memory tasks, and RBRs (*N* = 454).

	Yield 1	Yield 2	Shift	Total suggestibility	Delayed suggestibility
IR	–0.308***	–0.198***	–0.276***	–0.366***	–0.074
DR	–0.190***	–0.190***	–0.135**	–0.185***	–0.236***
Confabulation on IR	0.056	0.083	0.039	0.060	0.158**
Confabulation on DR	0.061	–0.020	–0.036	0.019	0.181***
Yield 1	–	0.509***	0.283***	0.821***	0.224***
Yield 2	0.509***	–	0.261***	0.488***	0.122*
Shift	0.283***	0.261***	–	0.779***	0.049
Total suggestibility	0.821***	0.488***	0.779***	–	0.177***
Delayed suggestibility	0.224***	0.122*	0.049	0.179***	–
NO 1	–0.403***	–0.154**	–0.183***	–0.372***	–0.108*
DE 1	–0.698***	–0.399***	–0.225***	–0.589***	–0.123*
DK 1	–0.330***	–0.162**	0.043	–0.189***	–0.049
NO 2	–0.232***	–0.227***	–0.208***	–0.277***	–0.061
DE 2	–0.612***	–0.469***	–0.324***	–0.592***	–0.152**
DK 1	–0.338***	–0.195***	–0.028	–0.236***	–0.041

**p*<0.05; ***p*<0.01; ****p*<0.001; IR, immediate recall; DR, delayed recall; DE 1 and 2, direct explanation answers; DK 1 and 2, don’t know answers.
